# Temperature Performance Study of SAW Sensors Based on AlN and AlScN

**DOI:** 10.3390/mi14051065

**Published:** 2023-05-17

**Authors:** Hui Wang, Linwei Zhang, Zhixin Zhou, Liang Lou

**Affiliations:** 1 School of Microelectronics, Shanghai University, Shanghai 201800, China; 2 The Shanghai Industrial μTechnology Research Institute, Shanghai 201899, China

**Keywords:** SAW sensor, AlN film, AlScN film, temperature performance

## Abstract

In this paper, the temperature performance of AlN-SAW resonators and AlScN-SAW resonators is studied. They are simulated by COMSOL Multiphysics, and their modes and the S11 curve are analyzed. The two devices were fabricated using MEMS technology and tested using VNA, and the test results were consistent with the simulation results. Temperature experiments were carried out with temperature control equipment. With the change in temperature, the changes in S11 parameters, TCF coefficient, phase velocity, and quality factor Q were analyzed. The results show that the temperature performance of the AlN-SAW resonator and the AlScN-SAW resonator is very good, and both have good linearity. At the same time, the sensitivity of the AlScN-SAW resonator is greater by 9.5%, the linearity is greater by 15%, and the TCF coefficient is greater by 11.1%. The temperature performance is excellent, and it is more suitable as a temperature sensor.

## 1. Introduction

A surface acoustic wave (SAW) is an acoustic wave that propagates on a solid surface, and its propagation speed is much lower than that of an electromagnetic wave, which is convenient for processing acoustic signals [1]. In recent years, SAW technology has developed rapidly and has been applied in many fields. For example, in high-voltage switchgear, its internal structure is complex, and it is in a closed environment. Under working conditions, high voltage and high current cause temperatures to rise, and the continuous rise in temperature may cause fire or even explosion [2,3,4]. The use of SAW sensors for temperature monitoring can reduce the accident rate. Excessive temperature of the train brake disc affects the train’s brake system. A SAW sensor can be installed on the brake disc for temperature acquisition [5]. In aerospace, SAW sensors can be installed inside aircraft engines to detect physical quantities such as temperature and pressure [6]. SAW devices have outstanding advantages: they do not require a wired connection, do not require battery power, and only communicate through the transceiver. At the same time, they are small in size, low in cost, do not require manual maintenance, and are easy to integrate [7].

Usually, we use piezoelectric materials to fabricate SAW devices. Piezoelectric materials are generally divided into three categories: piezoelectric single crystal, piezoelectric ceramic, and piezoelectric film. There have been a lot of studies on SAW temperature sensors based on piezoelectric materials. For example, Shu et al. reported a high-temperature strain sensor based on SAW that uses lanthanum gallium silicate (LGS) as its substrate, combines with a dipole antenna to form a wireless sensor, and conducts temperature tests in the range of 20 °C to 600 °C [8]. Wen et al. reported a SAW temperature sensor with lithium niobate (LiNbO_3_) as the substrate, and the temperature measurement experiment was conducted at 1100 °C with good repeatability and stability [9]. Sergei Zhgoon et al. reported a SAW temperature sensor based on a quartz substrate. Resonators with different cut shapes were designed, and TCF on a single substrate could exceed 135 ppm/°C [10]. A. Maskay et al. reported an LGS temperature sensor using Pt/Al_2_O_3_ as an electrode and tested its stability. The temperature was circulated for 110 h at 300 °C to 750 °C, and the frequency offset was 0.05% under six tests at 750 °C [11]. However, the traditional quartz material attenuates the sound speed and change the phase after exceeding a certain temperature, which makes it no longer suitable for temperature measurement [12]. For lithium niobate (LiNbO_3_) material, although it has a high mechanical and electrical coupling coefficient (K2), its temperature stability is relatively poor; at the same time, its chemical composition changes when the temperature exceeds a certain level [12]. Lanthanum gallium silicate (LGS) materials are widely used in high-temperature fields, but their surface acoustic velocity is low, and the acoustic propagation loss increases with the increase in temperature [13]. Aluminum nitride (AlN) material has a very high surface wave velocity [14], good linearity, and good temperature stability. Moreover, the preparation of the device can be compatible with the traditional silicon process, which has good potential in the field of temperature measurement [15]. AIN-based scandium admixture (AlScN) can also show good performance [16,17]. The comparison between the two and the above materials is shown in Table 1.

Among the above piezoelectric materials, AlN materials have good advantages in sound velocity, temperature stability, and material properties, especially in linearity. AlN material is doped with scandium, which can improve its temperature performance compared with AlN material. In order to explore how much the performance of the AlScN-SAW resonator can be improved compared with the AlN-SAW resonator in the aspects of linearity, stability, temperature, and frequency coefficient (TCF), this paper studies the SAW sensors of AlN and AlScN thin films. Firstly, they were simulated by COMSOL Multiphysics, and the working frequency was determined. Then, the MEMS process was used for fabrication. Finally, a temperature measuring platform was built for the temperature experiment, and the linearity, temperature frequency coefficient (TCF), frequency offset, repeatability, and other aspects were discussed.

## 2. Materials and Methods

### 2.1. Temperature Measurement Principle of SAW Sensor

The wave velocity of a surface acoustic wave is determined by the physical characteristics of piezoelectric materials. According to the perturbation theory [18], the wave velocity of a surface acoustic wave device is shown in Formula (1).

(1) $$\frac{\Delta V_{R}}{V_{R}}=-K_{m}\frac{\Delta m}{m_{0}}+K_{s}\frac{\Delta S}{S_{0}}+K_{\delta }\frac{\Delta \delta }{\delta }+K_{\gamma }\frac{\Delta \gamma }{\gamma }-K_{T}\frac{\Delta T}{T_{0}}+\ldots$$

where $V_{R}$ is the velocity of the surface acoustic wave, m is the mass loading on the piezoelectric surface, *S* is the stiffness, δ is the stress, γ is the surface tension, *T* is the temperature, $K_{m}$ is the mass sensitivity coefficient, $K_{s}$ is the stiffness sensitivity, $K_{\delta }$ is the stress sensitivity, $K_{\gamma }$ is the tension sensitivity, and $K_{T}$ is the temperature sensitivity.

According to Formula (1), the parameter $V_{R}$ is a function of temperature *T*, which is then converted by Taylor’s formula:

(2) $$V_{0}(T)=V_{R}(T_{0})[1+\frac{1}{V_{R}(T)}\frac{\partial V_{R}}{\partial T}(T-T_{0})+\frac{1}{2V_{R}(T_{0})}\frac{\partial^{2}V_{R}}{\partial^{2}T}{\left(T-T_{0}\right)}^{2}]$$

Finally, the velocity of the surface acoustic wave is completely determined by the temperature *T*:

(3) $$\frac{\Delta f}{f}=\frac{V_{R}(T)-V_{R}(T_{0})}{V_{R}(T_{0})}=\frac{1}{V_{R}(T_{0})}\frac{\partial V_{R}}{\partial T}(T-T_{0})$$

**Figure 1:**
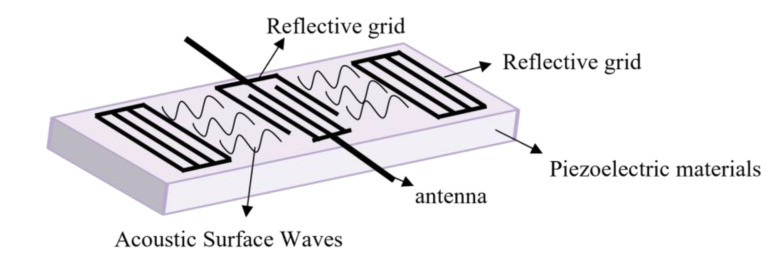
Piezoelectric effect of a surface acoustic wave device.

Therefore, according to Formula (3), the center frequency of the SAW device has an approximate linear relationship with the change in temperature. The center frequency is a function of temperature, and one piece of temperature data corresponds to one frequency, so temperature measurement can be completed. As shown in Figure 1, the interfinger transducer (IDT) uses the inverse piezoelectric effect to complete the electro-acoustic conversion. As the temperature changes, the speed of the sound waves changes. When IDT completes the acoustic-electrical conversion through the piezoelectric effect, the electromagnetic wave also changes and has temperature characteristics [19,20].

### 2.2. Simulation Model of the Sensor

**Figure 2:**
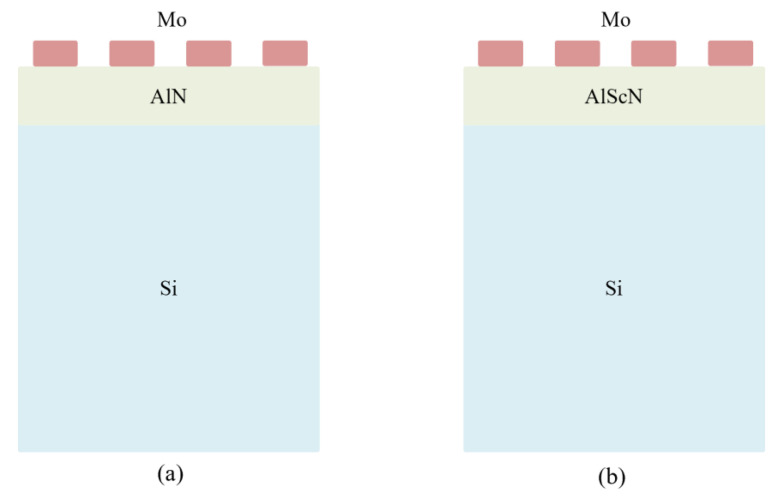
Model structure. (**a**) AlN-SAW resonator model structure. (**b**) AlScN-SAW resonator model structure.

The AlN-SAW resonators and AlScN-SAW resonators in this paper adopt a single-port double-finger IDT structure, which consists of an IDT and two reflector gates. Their top layer is a molybdenum electrode (Mo), the piezoelectric materials are AlN film and AlScN film, respectively, and the substrate is silicon material (polycrystalline silicon), as shown in Figure 2. Figure 2a is the structure of an AlN-SAW device, and Figure 2b is the structure of an AlScN device. The electrode thickness is 0.2 μm, the width is 1.2 μm, the thickness of AlN and AlScN is 1 μm, the acoustic wave wavelength is 9.6 μm, and the interfinger spacing is 1.2 μm.

COMSOL Multiphysics can realize the modeling of periodic infinite structures, so the finite element model was selected for this paper. The IDT structure is periodic, so the single IDT periodic unit can simulate the SAW resonator completely. When the surface acoustic wave propagates downward, it basically disappears after several wavelengths, so the height of the Si substrate was set to 2–3 times the wavelength, and the thickness of the Si substrate in this paper was 20 μm. The length of the IDT electrode is greater than its width, so there was no need to consider the edge effect of the electrode. Since the model in this paper is a periodic unit, periodic boundaries were set on both sides of the model, and low reflection boundaries were set at the bottom of the model. In the simulation of SAW resonators, the mechanical, electrical, and piezoelectric properties of materials should be considered. For AlN and AlScN layers, piezoelectric materials in solid mechanics and electrostatics were selected. The material parameters of the AlN layer, Mo electrode, and Si substrate are specified in the COMSOL library, and Sc doping in the AlN layer changes the AlN density and affects various piezoelectric coefficients of the AlN material. Table 2 shows the physical constants of each material. In order to compromise the calculation time and accuracy, finer grids should be set for the Mo electrode and AlN (AlScN) film, and the grids of the Si substrate can meet the minimum simulation requirements.

### 2.3. Fabrication

**Figure 3:**
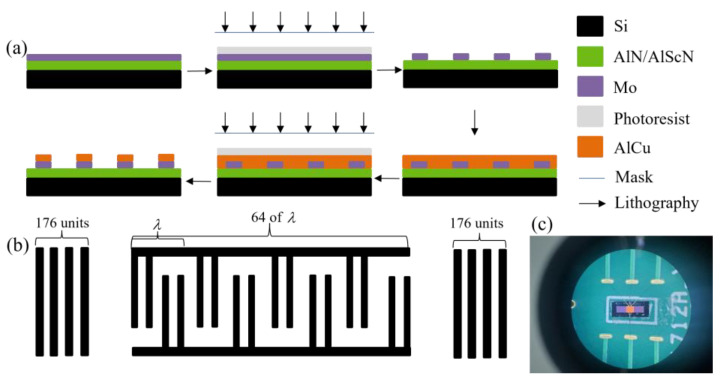
Fabrication of the SAW resonator. (**a**) The fabrication processes. (**b**) The reflector gate and IDT of the resonator. (**c**) The physical picture of the SAW resonator.

The fabrication process of SAW resonators is generally divided into three phases: substrate pretreatment, deposition of piezoelectric materials and electrodes, and lithography, as shown in Figure 3a. Because the substrate produces impurities due to various factors, the substrate needs to be cleaned. A clean substrate reduces the acoustic loss of the resonator and also makes the subsequent IDT and reflection grid electrode more adsorptive. After substrate pretreatment, AlN (AlScN) film (1 μm) and Mo electrode (0.2 um) were deposited. There are many deposition methods in SAW preparation, and magnetron sputtering technology was selected for this paper. For AlScN films, 20% scandium was doped on the basis of AlN films. The film was deposited on a polycrystalline silicon wafer by reactive radio frequency (RF) magnetron sputtering in a mixture of Ar and N_2_ gases. The target materials were pure aluminum and aluminum–scandium alloys with scandium doping concentrations of 20% and 20%, respectively. The concentration of the pure aluminum target was 99.99%, the purity of the aluminum–scandium alloy was 99.95%, and the diameter of both was 332 mm. Before insertion into the chamber, the silicon wafers were ultrasonically cleaned in acetone and alcohol successively for 8 min, then rinsed in deionized water for 5 min. The chamber was pumped to a base pressure of $10^{-8}$ Torr using a fast regen cryopump pump before argon and nitrogen were introduced. During deposition, the total gas flow rate of argon and nitrogen was maintained at 78 standard cubic centimeters per minute, the pressure in the chamber was maintained at $10^{-3}$, and the substrate temperature was maintained at about 302 °C. Other sputtering parameters, such as sputtering power (135 W) and nitrogen concentration (volume ratio of N_2_ volume to N_2_+Ar total gas volume), were adjusted to the required conditions to ensure a high C-axis orientation and better film thickness uniformity. The deposition time was varied to obtain a film with a thickness of about 1 μm. The substrate was processed by photolithography, and after the photoresist was spin-coated, the electrode pattern was formed on the substrate using a mask plate. After etching the photoresist, the Mo electrode was revealed. A layer of AlCu material (1 μm, deposited only on the pad) was deposited on the device for better connection with the metal wire. After the photolithography was completed, the SAW resonator based on AlN and AlScN was prepared. As shown in Figure 3b, the SAW resonators designed in this paper were all single-port, two-finger IDT structures, in which the IDT has a total of 64 cycles and each cycle has 4 cells. The reflection grid on each side of the IDT has 176 units. The size of the AlN-SAW resonator is exactly the same as that of the AlScN-SAW resonator. The only difference is that the AlN material is doped with scandium elements, so their physical pictures are the same under a microscopic image, as shown in Figure 3c.

### 2.4. Test Platform

**Figure 4:**
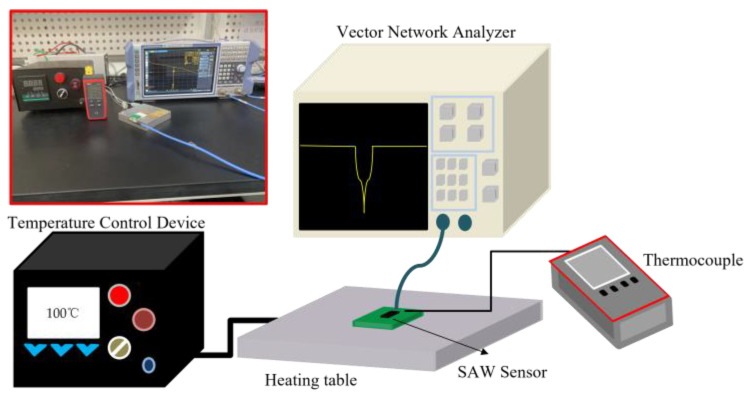
Establishment of a temperature measuring platform.

The temperature test platform is shown in Figure 4. The platform consists of a vector network analyzer (VNA, R&S^®^ ZNL/ZNLE Vector Network Analyzers) that comes from Rhodes & Schwartz (Munich, Germany), a temperature control device and a cast aluminum heating plate that come from Ruixin Electric Heating Company(Taizhou, China), a thermocouple (UT320A) that comes from Kepson Company (Jiangsu, China), and a SAW device. The prepared AlN-SAW resonator and AlScN-SAW resonator were placed on the PCB board, and the high-temperature thermal conductivity adhesive was used for bonding. The high-temperature, heat-conductive glue made the PCB temperature spread to the SAW resonator faster. Due to the power of the heating plate, the heating speed was fast, and if the heat dissipation speed of the heating plate cannot keep up with the heating speed, it is easy to make the aluminum material burn and melt. Therefore, the cast aluminum heating plate needed to be equipped with a temperature control device, and the thermite plate can withstand a working temperature of about 350 °C. In the actual test, although the temperature control device could set a certain temperature, there is a large error between its displayed temperature and the actual temperature. Therefore, the thermocouple device was used to calibrate the temperature. The precision of the thermocouple is about 0.1 °C, which can effectively reduce the experimental error. The diameter of the thermocouple probe was about 0.1 mm, and the temperature range was below 200 °C. The temperature of each point on the PCB board may have been different to some extent. The probe position was attached to the nearest SAW resonator on the PCB board to reduce the test error. The SAW resonator was connected to the VNA through a coaxial cable, which can operate at high temperatures. Considering the temperature resistance of PCB board, the highest temperature tested in this paper was 180 °C. Before the temperature measurement experiment, we opened VNA and stood for a few minutes, and then used the standard calibration part to calibrate it so that the open, short, match, and through ports of the calibration part were matched with the ports of VNA, respectively.

## 3. Results and Discussion

### 3.1. Reflection Coefficient S11

**Figure 5:**
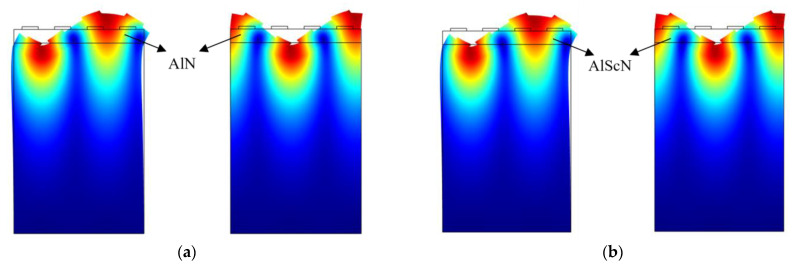
Mode of SAW resonator. (**a**) The symmetry and antisymmetric mode of the AlN-SAW resonator. (**b**) The symmetry and antisymmetric mode of the AlScN-SAW resonator.

In the simulation of AlN-SAW resonators and AlScN-SAW resonators, the characteristic frequencies and the mode shapes were obtained the eigenvalues study in COMSOL. Studies were added to COMSOL, and the study content was characteristic frequency analysis. Among them, the characteristic frequency solver was ARPACK, the number of characteristic frequency searches was 10, and the search reference value was 500 MHz. After the calculation of the characteristic frequency analysis, the mode shape analysis was selected among the study results. Finally, the correct perturbation state was selected from 10 characteristic frequency data points. Figure 5 shows their symmetric mode and antisymmetric mode comparison; the symmetric mode was generated by the perturbation of the real mode, and the antisymmetric mode was generated by the perturbation of the complex mode. Two modes of the AlN-SAW resonator were found at 502 MHz; Figure 5a shows the symmetric and antisymmetric mode of the AlN-SAW resonator. Two modes of the AlScN-SAW resonator were found at 493 MHz; Figure 5b shows the symmetric and antisymmetric mode of the AlScN-SAW resonator. 

**Figure 6:**
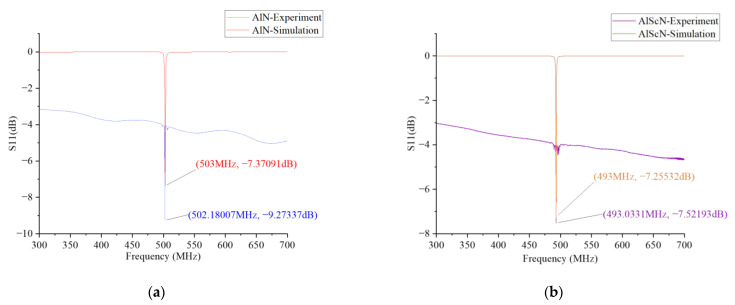
Simulation and test of the S11 curve of the AlN-SAW resonator and the AlScN-SAW resonator. (**a**) simulation and test results of AlN-SAW resonator S11. (**b**) simulation and test results of AlScN-SAW resonator S11.

The modeled S11 parameters were obtained with the frequency domain analysis in COMSOL. We determined the sweep frequency and sweep step size in the frequency domain analysis. The scanning frequency ranged from 300 MHz to 700 MHz. The scanning step was 0.1 MHz. After the calculation of frequency domain analysis, one-dimensional plot groups and globals were selected from the results. The global data set selected the data studied in the frequency domain. The Y-axis data were derived from the S11 parameter in the terminal. The SAW resonator was tested using VAN. Figure 6 shows the simulation and test comparison of the AlN-SAW resonator and the AlScN-SAW resonator at room temperature (25 °C). In the simulation, the resonant frequency of the AlN-SAW resonator was 503 MHz, and S11 was −7.37091 dB The measured resonant frequency was 502.18007 MHz, and S11 was −9.27337 dB, as shown in Figure 6a. The simulated resonant frequency of the AlScN-SAW resonator was 493 MHz and −7.255 dB for S11, while the measured resonant frequency was 493.0331 MHz and −7.52193 dB for S11, as shown in Figure 6b. The difference between simulation and testing was caused by the mismatch between the density and elastic matrix of the two piezoelectric materials and the material parameters in simulation. 

**Figure 7:**
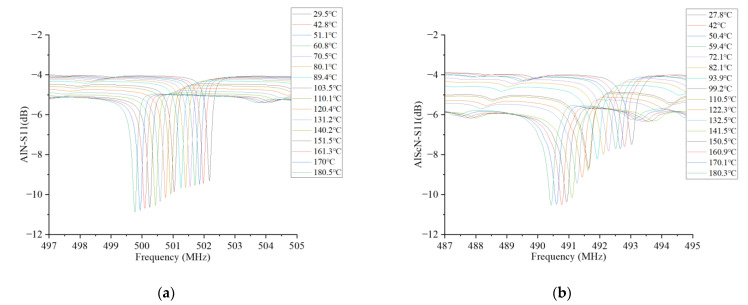
The S11 curves of the AlN-SAW and AlScN-SAW resonators as a function of temperature. (**a**) Test results of AlN-SAW resonator S11 as a function of temperature. (**b**) Test results of AlScN-SAW resonator S11 as a function of temperature.

After testing the S11 parameters of two kinds of resonators, the relationship between their S11 curves and temperature was studied. The temperature from 29.5 °C to 180 °C was recorded every 10 °C or so. Figure 7 shows the relationship between S11 and temperature. It can be seen that with the increase in temperature, the resonance frequency of both reduced, while at the same time, |S11| increased. The increase in |S11| made the loss of the signal lower and the signal quality better. A rise in temperature changed the thickness and density of the two materials, which affected wave speed, which is one reason for the decrease in frequency.

### 3.2. Phase Velocity

**Figure 8:**
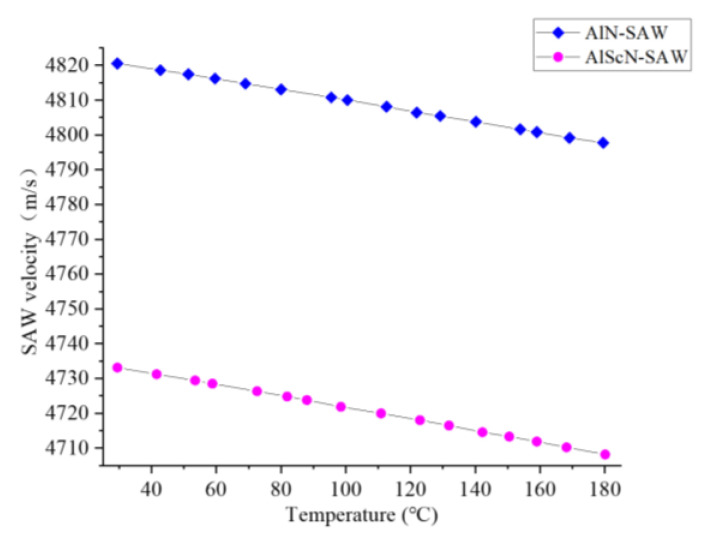
Phase velocity as a function of temperature for AlN-SAW resonators and AlScN-SAW resonators.

The phase velocity of the SAW resonator is defined by Equation (4):

(4) v=f×λ

where *f* is the resonant frequency of the SAW resonator and *λ* is the wavelength of the acoustic surface. Figure 8 shows the test results of the phase velocities of the two.

At room temperature, the phase velocity of the AlN-SAW resonator was 4820 m/s, and that of the AlScN-SAW resonator was 4733 m/s. The lower elastic constant of the AlScN-SAW resonator is one reason for its lower phase velocity. The test results of the phase velocities of the two, and the change in their phase velocities with temperature can be studied. As the temperature increased, the phase velocity of the two decreased to a certain extent.

### 3.3. Temperature–Frequency Relationship

The temperature–frequency relationship is generally expressed by the temperature–frequency coefficient (TCF). Formula (5) defines TCF, expressed in ppm/°C:

(5) $$TCF(ppm/℃)=\frac{1}{\Delta T}\frac{f_{\max}-f_{\min}}{f_{\min}}\times 10^{6}$$

where ΔT is the temperature measuring range, $f_{\max}$ is the frequency value corresponding to the highest temperature in the temperature measuring range, and $f_{\min}$ is the frequency value corresponding to the lowest temperature in the temperature measuring range.

**Figure 9:**
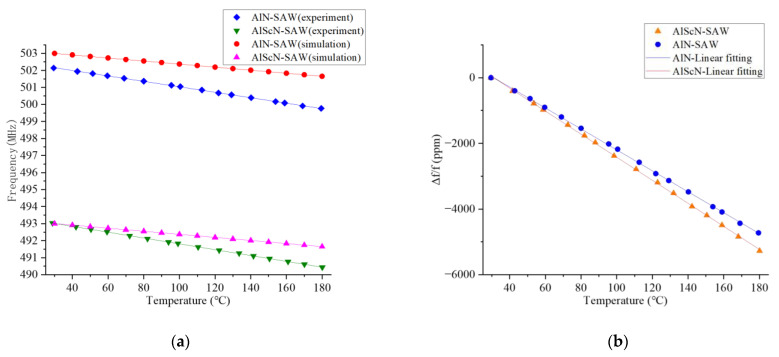
(**a**) The variation in the resonant frequency with temperature. (**b**) Relative evolution of the resonant frequency as a function of temperature.

The test results of the relationship between temperature and frequency are shown in Figure 9. Temperature points and frequency points are represented by an XY coordinate graph, and the fitting curve was generated by linear fitting. The simulation and test results show that both of them have good linearity and frequency response. There were some differences between the parameters of the materials in the simulation and those in the preparation, which is one of the reasons for the errors in the simulation and testing. The frequency of the AlN-SAW resonator varied by 2.37 MHz in the temperature range of 29.5 °C to 179.5 °C, and the sensor sensitivity was 15.8 kHz per °C. Through the calculation of the fitting curve and each point, the average linear error was 0.75 °C, and the linearity was 1.18%. The TCF coefficient calculated by formula 4 was −31.51 ppm/°C. The frequency of the AlScN-SAW resonator changed by 2.600078 MHz in the temperature range of 29.5 °C to 180.1 °C, the sensor sensitivity was 17.3 kHz/°C, the average linear error was 0.75 °C, the linearity was 1%, and the TCF coefficient was −35.02 ppm/°C. With 20% scandium doped in the AlN material, the sensitivity of the AlScN-SAW resonator was higher than that of the AlN-SAW resonator, which increased by 9.5%. It showed better linearity, up by 15%; the absolute value of the TCF coefficient became larger, increasing by 11.1%. It can be seen that the temperature performance of the AlScN material is better than that of the AlN material.

**Figure 10:**
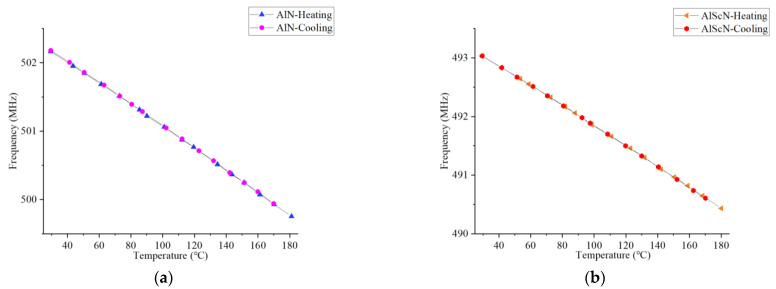
Return curve of the AlN-SAW and AlScN-SAW resonators. (**a**) Return curve of the AlN-SAW resonators. (**b**) Return curve of the AlScN-SAW resonators.

In order to study the frequency migration characteristics of the two, a return curve was tested in the temperature range of 29.5 °C to 180 °C, as shown in Figure 10a. Figure 10a shows the return curve of the AlN-SAW resonator. In general, it can be seen that its heating–cooling curve was basically consistent, but the frequency was shifted by 26.7 kHz when it was cooled to 29.5 °C. Figure 10b shows the return curve of the AlScN-SAW resonator, which had the same heating and cooling curve characteristics as AlN, while there was no frequency offset. In wireless temperature measurement, a frequency offset may lead to inaccurate temperature detection. The frequency shift in the AlN-SAW resonator may be related to the AlN material. The stiffness of the AlN material was stronger than that of the AlScN material, and the deformation was not easy to recover after cooling, resulting in certain frequency errors after cooling.

**Figure 11:**
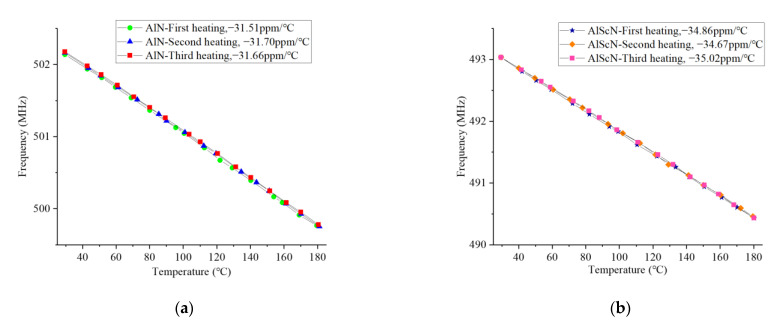

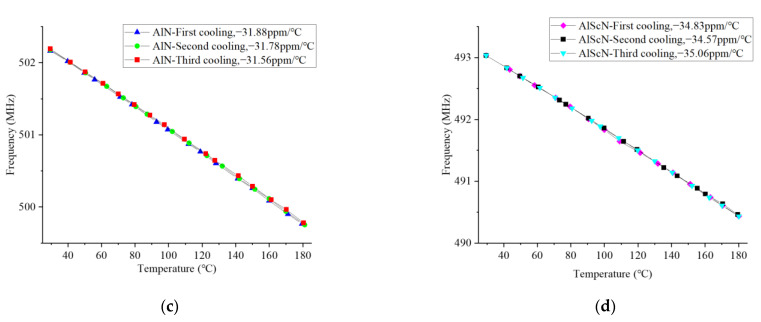
Three heating and cooling of the AlN-SAW (**a**,**c**) and AlScN-SAW resona-tors (**b**,**d**).

The cast aluminum heating plate was used to heat up and cool down the two materials three times, and the test results are shown in Figure 11. Figure 11a,c show the cubic temperature rise curves of the two. In the three heating periods, the TCF coefficients of the AlN-SAW resonator were −31.51 ppm/°C, −31.7 ppm/°C, and −31.66 ppm/°C, respectively. The TCF coefficients of the AlScN-SAW resonator were: −34.86 ppm/°C, −34.67 ppm/°C, and −35.02 ppm/°C. Figure 11b,d show the three cooling curves of the two. The TCF coefficients of the AlN-SAW resonator were −31.88 ppm/°C, −31.78 ppm/°C, and −31.56 ppm/°C, respectively. The TCF coefficients of the AlScN-SAW resonator were: −34.83 ppm/°C, −34.57 ppm/°C, and −35.06 ppm/°C. We can see that both of them have good repeatability.

### 3.4. Quality Factor Q

**Figure 12:**
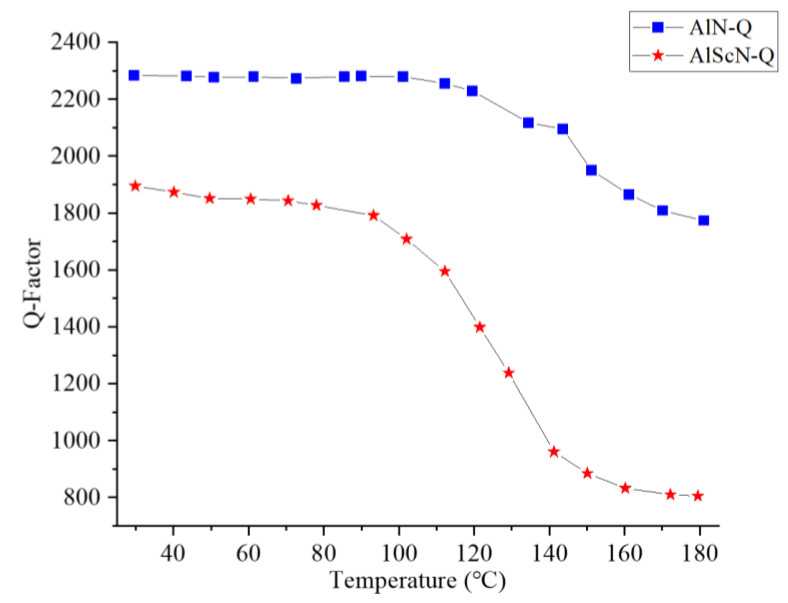
Quality factor *Q* of the AlN-SAW resonator and the AlScN-SAW resonator as a function of temperature.

The quality factor *Q* generally represents the energy storage of SAW devices, which is defined by Equation 6:

(6) $$Q=\frac{f_{0}}{BW(-3\;\mathrm{dB})}$$

where $f_{0}$ is the resonant frequency of the SAW resonator and BW (−3 dB) is the −3 dB bandwidth of S11. As shown in Figure 12, the relationship between quality factor *Q* and temperature was studied. As can be seen from Figure 12, the quality factor of both decreased with the increase in temperature, and less energy was stored in the SAW devices. The quality factor *Q* of the AlScN-SAW resonator was lower than that of the AlN-SAW resonator, which will be improved in future work.

## 4. Conclusions

In this paper, AlN-SAW resonators and AlScN-SAW resonators were prepared by depositing AlN films and AlScN films on Si substrate. Their temperature performance was studied from S11 parameters such as phase velocity, TCF coefficient, and *Q* value. The experimental results show that their S11 curve, phase velocity, and *Q* value decreased with the increase in temperature, and their resonant frequency transformed linearly with temperature. The sensitivity of the AlN-SAW resonator was 15.8 kHz/°C, the mean linear error was 0.75 °C, the linearity was 1.18%, and the TCF coefficient was −31.51 ppm/°C. The sensitivity of the AlScN-SAW resonator was 17.3 kHz/°C, the mean linear error was 0.75 °C, the linearity was 1%, and the TCF coefficient was −35.02 ppm/°C. Compared with the AlN-SAW resonator, the sensitivity of the AlScN-SAW resonator increased by 9.5%, the linearity increased by 15%, and the TCF coefficient increased by 11.1%; its temperature performance is better, making it more suitable as a temperature sensor. In future work, we will further increase their *Q* values so that the resonator reserves more signal energy.

## Figures and Tables

**Table 1:** The comparison between AlN, AlScN, quartz material, LiNbO_3_, and LGS.

Material	Temperature-Sensing Characteristic	Advantages	Disadvantages
AlN/AlScN	Electrical signals have a linear relationship with temperature. Good temperature-sensing characteristics.	Higher wave speed. Compatible with conventional silicon technology. Applicable to high-frequency field.	Film-growing technology is complex.
Quartz material	The temperature-sensing characteristics are linear. Low sensitivity.	Good linearity.	Large acoustic attenuation. Low-phase transition temperature.
LiNbO_3_	High sensitivity at lower temperatures.	High electromechanical coupling coefficient.	The temperature stability is poor.
LGS	High temperature-sensing sensitivity. Large electrical signals are generated when the temperature changes.	High-temperature field is widely used.	Low surface acoustic velocity. Increasing acoustic propagation losses with the temperature at higher frequency.

**Table 2:** Physical constants of AlN, AlScN [21], Mo, and Si.

Material		AlN	AlScN	Si	Mo
Density (kg/m^3^)	ρ	3260	2614	2320	10,200
Elastic constants (GPa)	$C_{11}$	410	328	-	-
	$C_{12}$	137	149	-	-
	$C_{13}$	99	111	-	-
	$C_{14}$	-	-	-	-
	$C_{33}$	389	311	-	-
	$C_{44}$	125	100	-	-
Piezoelectric constants (C/m^2^)	$e_{15}$	−0.48	−0.38	-	-
	$e_{31}$	−0.58	−0.69	-	-
	$e_{33}$	1.55	1.86	-	-
Dielectric constant (10^−11^ F/m)	$\varepsilon_{11}$	9	10.8	4.5	11.7
	$\varepsilon_{33}$	11	13.2	-	-
Thermal expansion coefficient (1/°C × 10^−6^)	α	4.5	4.5	2.6	4.8
Dielectric loss (×10^−3^)	δ	2			
Mechanical loss (×10^−3^)	η	4			

## Data Availability

Not applicable.
